# Human Autonomy in Future Drone Traffic: Joint Human–AI Control in Temporal Cognitive Work

**DOI:** 10.3389/frai.2021.704082

**Published:** 2021-07-20

**Authors:** Jonas Lundberg, Mattias Arvola, Karljohan Lundin Palmerius

**Affiliations:** ^1^ Department of Science and Technology, Linköping University, Norrköping, Sweden; ^2^ Department of Information and Computer Science, Linköping University, Linköping, Sweden

**Keywords:** human-centered AI, autonomy, scenario-based design, unmanned traffic management, joint control framework, interaction design, visualization design, UTM

## Abstract

The roles of human operators are changing due to increased intelligence and autonomy of computer systems. Humans will interact with systems at a more overarching level or only in specific situations. This involves learning new practices and changing habitual ways of thinking and acting, including reconsidering human autonomy in relation to autonomous systems. This paper describes a design case of a future autonomous management system for drone traffic in cities in a key scenario we call The Computer in Brussels. Our approach to designing for human collaboration with autonomous systems builds on scenario-based design and cognitive work analysis facilitated by computer simulations. We use a temporal method, called the Joint Control Framework to describe human and automated work in an abstraction hierarchy labeled Levels of Autonomy in Cognitive Control. We use the Score notation to analyze patterns of temporal developments that span levels of the abstraction hierarchy and discuss implications for human-automation communication in traffic management. We discuss how autonomy at a lower level can prevent autonomy on higher levels, and vice versa. We also discuss the temporal nature of autonomy in minute-to-minute operative work. Our conclusion is that human autonomy in relation to autonomous systems is based on fundamental trade-offs between technological opportunities to automate and values of what human actors find meaningful.

## Introduction

Industry is currently in a race to build and deploy autonomous vehicles in all areas of transport. At the same time, concepts for controlling and coordinating the traffic systems in which they operate are also developed. There are good reasons to believe that those control systems also will be autonomous systems. The label *autonomous system* is today given to a technological system that can manage itself to some degree. An autonomous system can impede *human autonomy,* which refers to the freedom to reason without constraints from authority and preconceptions (Stensson and Jansson, 2014). Artificial intelligence (AI) will give rise to new complementary roles of autonomous systems and human autonomy, and human responsibilities will shift. At the limits of what artificial intelligence can do, various human roles are invented, such as the human in the vehicle or the human in the control center with the role of taking over if an automation fails. This question of what human roles the system requires, or that are desirable from human points of view, is still a current topic. What we know is that the introduction of automation in vehicles or whole transport systems means that human autonomy is affected–in various ways. The questions are how and at what level (from deciding what the situation is about, to specific actions). In this paper we explore human autonomy and humans’ ability to leverage the automation for their own aims. This includes controlling, taking over, or guiding the activities of those systems, in part, or in full. We will argue that a system design must consider the level of autonomy of the automation, the autonomy of the human, and the autonomy of the joint system, as a situation develops dynamically.

The human ability to understand and control other systems, with a focus on dynamic mission critical systems, has been studied extensively in Cognitive Engineering. It builds upon notions of distributed cognition (Hollan et al., 2000), situated action (Suchman, 1987), and cybernetics (Ashby, 1956; Mantovani, 1996). Cognitive Engineering studies interaction for control of and control in such systems (Hollnagel and Woods, 1983; Rasmussen, 1986; Vicente, 1999). A contribution from the area has been a set of Cognitive Work Analysis (CWA) and Cognitive Systems Engineering (CSE) methods. A particularly useful notion from CWA for the purpose of this study is levels of abstraction (Rasmussen, 1986; Flach, 2017), or layers of control (Hollnagel and Woods, 1983). It is a means of describing and analyzing the system to be designed across different levels of abstraction ranging from *why* (effect goals and targets) and *what* the system does and achieves (qualities/performance levels, plans/blueprints), to *how* it carries it out in particular situations and instances (objects, actions, structures). These abstraction levels or control layers can be analyzed over time (Lundberg and Johansson, 2020), as a hierarchical system (Read et al., 2015; Naikar, 2017), as situated control actions (Hollnagel and Woods, 1983), as part of a semiotic system (Flach, 2017), or in terms of resilience (Lundberg et al., 2012). However, in this paper we are concerned with how AI-human interactions on these levels affects understanding, action, and choice as a central part of human autonomy. Thus, we discuss autonomy considering long-standing concerns in human-machine interaction and communication. This includes the narrowing down of choice through framing (Schön, 1983), generation vs. selection of alternatives, recurrence vs. uniqueness of breakdowns, resolution as conversation (Winograd and Flores, 1986; Schön, 1992), situatedness vs. planning and ad-hoc solutions (Suchman, 1987), opportunities and interests vs. tools and users, and action and structure (Mantovani, 1996).

In this article, we aim to show how, on the one hand, simulation and visualization can facilitate analyses of and design for human autonomy in interaction with autonomous systems, and on the other hand, how to analyze temporal developments that span levels of the abstraction hierarchy. This contribution is of general relevance to the academic community as an approach to understanding human autonomy in collaboration with autonomous systems, and the discussions on unmanned traffic are of relevance to the growing applied area of autonomous transport. The article illustrates how this analysis can be conducted, and the outcomes of the analysis are discussed with a basis in scenarios co-designed with domain experts in air traffic management, and in simulated traffic situations from these scenarios.

## Materials and Methods

The research method was a case study (Runeson and Höst, 2009) of the simulation- and scenario-based design of an unmanned aircraft system (UAS) traffic management (UTM) system, including both unmanned traffic and regular traffic, with an analytic focus on human autonomy. The Levels of Autonomy in Cognitive Control (LACC) model of an abstraction hierarchy, from the Joint Control Framework (JFC) (Lundberg and Johansson, 2020), was used for the analysis of autonomy. This model has six levels going from why (framing and effects goal formation) and what (values/metrics and generic schemes), to how (implementation and physical interactions):

Level 6 (frames)–Framing the Situation: What kind of situation is it and what is our overarching approach? Different people will frame the situation differently and value different things. Unmanned vehicles might be about entertainment for one person, but for another person it might be about deliveries. There might be a pre-conceived framing of how things should be managed, and what must be done and controlled. This is a key decision with respect to autonomy, as it frames all the levels below, from goal formation to action. In many cases the frame is taken for granted, but it can also be a source of fundamental conflicts of interest.

Level 5 (effects)–Formulating Effect Goals: What qualities or dimensions in the situation is to be affected? Effect goals can, for instance be to achieve accessibility, safety, flexibility, or satisfaction. What are the desired effects on the situation or process under control? When we ask the question, “what is the purpose of the system?“, then we address level 5.

Level 4 (values)–Setting Values and Metrics: What is the range of possibilities? Within the range, what are the trade-offs between potentially conflicting effect goals, what are the high-level key performance indicators (KPIs), and what are their threshold values? Compared to level 5, this level is about the degree of achievement of goals, but also about performance indicators that relate to (but are not complete or direct measures of) the level 5 effect goals.

Level 3 (generic)–Choosing and Monitoring Generic Schemes: This level concerns the organization of functions. What are the overarching or generalized functions, plans, structures and patterns? What is the purpose of those schemes in relation to KPIs and effect goals on level 4 and 5? These are re-usable kinds of plans, such as the overall plan for a vehicle driver to overtake another vehicle. It also concerns the execution of the plan as a whole, and monitoring of progress.

Level 2 (implementation)–Determining Implementation and Execution: How should the generic schemes (functions, plans, patterns, and structures) be implemented and executed? What physical resources should be allocated? Specific implementations and executions (e.g., particular positioning systems or specific models of drones) come with constraints on the physical interaction on level 1, and they may also have side-effects impacting the effect goals on level 5. For example, a particular vehicle may have a particular acceleration limit, turn radio, and noise emissions.

Level 1 (physical)–Deciding Physical Interaction, and Object Status: What are the physical objects, their properties and their physical layout? What are the specific actions on physical objects, places, and associated constraints (e.g., resource limits for implementation)? In drone traffic this can involve for example steering, hazards in some physical areas, and the locations of things.

### Case Selection

Our case is UTM (unmanned aircraft systems traffic management) to control future intense air traffic in cities. This is an area where vehicles and traffic management systems are being developed currently. The case of UTM over cities was chosen based on the importance and potential impact of the case for the future of air traffic management as well as service development. We chose to work with cities and not rural areas because urban traffic is expected to place higher demands on traffic management. This makes increased system autonomy more interesting to pursue for the stakeholders. The introduction of light, electrical, unmanned aerial vehicles that can operate beyond line-of-sight and autonomously detect and avoid other traffic, opens the door to new high traffic intensity services. For an overview of services suggested by various stakeholders, see for instance Lundberg et al. (2018a).

UTM can benefit from highly autonomous traffic management, to overcome the limits of traditional approaches. In regular air traffic management, controllers manually monitor each aircraft, approving or declining requests for changes in altitude or direction, and make calls to pilots to adjust their trajectories as needed to avoid conflicts and to optimize traffic. Such traffic moves slowly over the operator’s screen due to the long distances involved and even so manual management is limited to about 7–15 regular aircraft simultaneously in a sector. In contrast, drones over a city would move relatively fast over the screen, due to the shorter distances, and potentially reach many possible conflicts over a short period of time. Because of this, the ATM approach has in previous research been seen as impractical for intense drone traffic, and a higher level of autonomy of the traffic management system was suggested (Lundberg et al., 2018a). With few drones, each drone can for instance be given its own dedicated flight level or volume, reducing the need for continuous monitoring for conflicts. However, with more intense traffic, a need to share airspace between drones and service providers arises, and decisions must be made on what approach to use (i.e., points, lines, layers, volumes or a combination) depending on the situation at hand. If management of traffic movements is delegated to an autonomous system, the manager gets more overarching tasks, such as overseeing restriction areas, contingency management, and traffic load (Lundberg et al., 2018b).

Within that overarching case we chose to focus on how UTM operators can optimize traffic when there are traffic conflicts between competing service operators. Such conflicts require the use of some kind of detect and avoid mechanism. This ad-hoc situation handling introduces a risk (upon sensor or algorithmic failure) and a disturbance in the timing and predictability of services, as well as requiring extra energy usage. However, de-confliction is critical for high density future traffic to work well. The input used for the initial problem framing was earlier analyses of stakeholder needs, simulation studies, and workshops with air traffic controllers, regulators, drone pilots, and service users (Lundberg et al., 2018a; Lundberg et al., 2018c; Halvorsen, 2018).

The rapid development of artificial intelligence (AI) makes it likely that each vehicle, service, and the management system itself could operate with a high degree of autonomy. However, we can assume that all of these operate based on the goals and desires of humans, rather than having their own agenda. Thus, a key question arises in the UTM case. How can human operators constrain, direct, and/or coordinate these systems? A first step toward answering that question is to be able to analyze emerging and existing designs from the perspective of human autonomy. Accordingly, in this paper, we focus on how we can describe such systems and situations from the perspective of human autonomy, to assess properties of emerging designs. To start addressing this question, in this paper we design and evaluate one larger scenario, and the temporal development of one episode from the scenario implemented in a drone traffic simulator.

### Drone Traffic Simulation

To facilitate empirical research on future drone traffic situations and management, a drone traffic and management simulator has been implemented. To ensure that simulated scenarios become realistic regarding separation between (potentially autonomous) units, the system makes strict separation between services, drones and control authorities, and simulates communication and negotiation between these units. The currently active services and simulated traffic are visualized together with 3D maps in an interface that functions both as a scenario design tool and a traffic management tool, through different menus. When a scenario designer has defined services (e.g., package delivery from a transport hub) the system simulates, for a randomized continuous stream of drones, these services making flight planning and negotiating with control authorities, and the subsequent flight authorization, take-off, flight and landing, according to specified traffic frequency and drone capability parameters associated with each service. A traffic manager can then add, move and remove geofences and adjust flight levels, and for each change in the airspace, the services are challenged to renegotiate their currently running plans.

Each autonomous drone is provided with sensory and network data and will activate a simple collision avoidance routine upon detecting proximity with other drones. Such conflicts can–for drones that follow an authorized plan to follow a path, such as delivery, some types of monitoring and other tasks that can be pre-planned–be predicted based on current position and speed, and these potential future conflicts can then be visualized for an overview of future problems of this regard. The system also provides fast-forward functionality, to facilitate quick experimentation with scenarios and conflicts resulting from changes in service characteristics and airspace configurations.

### Design and Analysis Procedures

The scenarios were developed by storytelling and sketching, with design questions and assessment of alternatives structured by LACC. The process followed a scenario-based design process (Carroll, 1995; 2000; Rosson and Carroll, 2002; Rosson and Carroll, 2008), extended with the use of HMI-T Score notation from the JCF (Lundberg and Johansson, 2020). Design expressed in scenarios were at different levels of abstraction, which follows earlier design cases (Arvola and Broth, 2019; Blomkvist and Arvola, 2014; Rosson and Carroll, 2002; Rosson and Carroll, 2008):

1) Problem analysis
2) Design of scenarios of future use situations.
3) Assessment of scenarios made in the form of claims.
4) Assessment of scenarios in a traffic simulation.
5) HMI-T Score analysis
  i) Process mapping
  ii) Laying out joints (perception, decision, action) on the Score
  iii) Dividing the Score into sub-episodes
  iv) Placing the joints on the correct LACC levels

The scenarios were specific narratives about hypothetical everyday use of systems. They were created to facilitate a dialogue on how new technologies fit into stakeholders’ everyday life and their ways of working. They were based on discussions in a workshop with four participants (Workshop 1). Three of them worked with air traffic control. Two of those were also airport managers, and the third worked with drone issues. The fourth participant, who worked with incident management and airport equipment, was also an experienced model plane flyer and hobby drone pilot. The workshop was recorded on video after informed consent from the participants. Fieldnotes were taken by two researchers. The workshop had an introduction to UTM by one of the authors, after which the current simulation of UTM traffic was demonstrated, and the participants set up geofences (no-fly areas) and observed how the traffic was affected. Emergent traffic situations, events and issues were then discussed, and potential solutions were suggested. The first step of the analysis of the workshop was to identify problem situations in the recordings by listening through and taking notes of all situations that seemed relevant. This generated a base document of about 4,000 words. The second step was to code problem situations in the base document, and the third step was to categorize the codes, and finally select what problem situations to include in the present iteration of development. The procedure of the analysis followed the six phases of thematic analysis identified by Braun and Clarke (2006): 1) Familiarizing with the data; 2) generating initial codes; 3) searching for themes; 4) reviewing themes; 5) defining and naming themes; and 6) producing the report. The levels in the LACC model were used to generate initial codes in a theory-driven top-down approach. The same approach was used to analyze claims made throughout the design and development process.

Five scenarios of future use situations where the identified problems could be managed by the air traffic control were then developed. Two scenarios focused on how air traffic control management worked during operations, and three focused on planning of the airspace before operations. So called, what-if scenarios supported the framing and re-framing necessary for radically new designs (Dorst, 2015; Ylirisku et al., 2016). The scenarios were prioritized by the research and development team, based on a combined assessment of feasibility and novelty. Scenarios were analyzed using claims analysis, which means that consequences of design alternatives were assessed in pro et contra lists (Rosson and Carroll, 2002; Rosson and Carroll, 2008). Design consequences that propagated across levels of control were noted (Moran, 1981; Buxton, 1983; Schön, 1992).

At a later stage, a selected key scenario for operations was assessed in a walkthrough held remotely in a video conference due to pandemic restrictions (Workshop 2). The selection of that scenario was made based on the claims analysis prepared from the first workshop. The walkthrough involved five experienced participants with a background in air traffic control. It started with a presentation of the scenario, followed by a demonstration of the simulation of urban drone traffic. The workshop was recorded and the parts relating to the scenario were transcribed in verbatim. Informed consent was obtained from the participants using an online form, since the workshop was held remotely over video conference, but information about the study was sent to the participants to read prior to the walkthrough.

**FIGURE 1:**
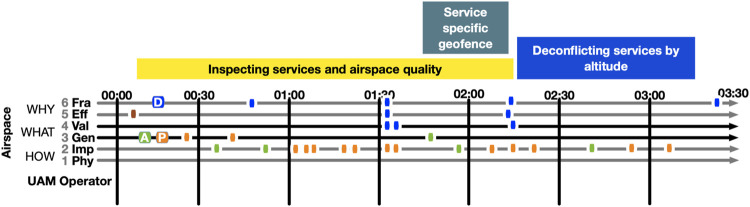
JCF Score of the scenario (see also the video recording in the Supplementary Material to this article). Vertically, the LACC shows the level of cognitive control. Horizontally, temporal developments are shown from left to right. The colored dots indicate perception (orange), decision (blue), action (green), and core values (red). What occurs at these positions in the Score is presented in Table 1.

Subsequently (step 5), the simulation was used to test, analyze, and refine scenarios further. We analyzed the temporal aspect of autonomy in a control episode. For this, we used the HMI-T Score notation from the JCF (Lundberg and Johansson, 2020), which lays out *joints* (perceptions, decisions and actions) between various subjects and objects, over time, on the LACC levels. The JCF Score describes the temporal flow of interaction between the operator (agent/subject) and the object process through the interface (to the autonomous system, automation, or physical means to affect and view the process). It is described as perception, decision, and action joints. In a simplified analysis, the temporal order is noted, but we also noted the exact timing of interactions based on a recording of the simulation of the process (Figure 1). Each joint is depicted as a discrete point in the Score, which also is a simplification. The analysis was conducted using our own research software for LACC analysis through four steps (i–iv).

In step *i*, the processes to focus on in the analysis were identified, determining the subject(s) and object(s) in the analysis. As discussed by Flach (2017), interaction occurs in a semiotic triad of agent, interface, and process. In JCF (Lundberg and Johansson, 2020), this relation is described as subject, interface, object/process. With an autonomous system working for a human operator, the human sets process-related goals and constraints for the automation. In our analysis, we have not analyzed the emergence of disturbances in the process, although in other cases this might be important. We have also excluded the internal mechanisms of the autonomous system, which might be relevant for other purposes. In the analysis of this paper, we focus on situations where a human supervises and controls an autonomous system. In this case (Figure 1), that resulted in one main traffic processes to describe using the Score notation.

In step *ii*, based on the simulated scenario, the joints were described by placing them in temporal order on the Score (Figure 1, and annotated, Table 1). The annotations describe the contents of each joint. In this step, it is not critical to get the points exactly at the right LACC level since that is fine-tuned in step *iv*. This step describes what is seen, decided, and done, and the timing of these joints.

**TABLE 1:** Temporal development of scenario fragment. See Figure 1 for a visualization and the video in the Supplementary Material to the article for a recording.

Time	Type	Level	
+00:00:09	CoreValue	Effects	Associated core value, effect goal– efficient airspace, reduce or eliminate conflicts
+00:00:13	ActionPoint	Generic	Adding planned package delivery services A and B for inspection
+00:00:16	DecisionPoint	Frames	The crossing service lines holds the potential for between-service conflicts
+00:00:16	PerceptionPoint	Generic	Observing the crossing service lines
+00:00:26	PerceptionPoint	Generic	Inspecting the generic plan for service A (speed, altitude, drones per minute)
+00:00:35	ActionPoint	Implement	Activating simulation of service A
+00:00:41	PerceptionPoint	Generic	Inspecting the generic plan for service B (speed, altitude, drones per minute)
+00:00:47	DecisionPoint	Frames	The observed use of the same altitudes for both services, reinforces the initial frame (risk of service conflicts), but is not decisive
+00:00:51	ActionPoint	Implement	Activating simulation of service B
+00:01:02	PerceptionPoint	Implement	Inspecting emerging simulated traffic
+00:01:06	PerceptionPoint	Implement	Activating fast forward to inspect fully developed service traffic
+00:01:08	PerceptionPoint	Implement	Observation of conflicts in the services (orange X)
+00:01:18	PerceptionPoint	Implement	Observing the location of conflicts and crossing trajectories over hubs and in crossing traffic
+00:01:22	PerceptionPoint	Implement	Inspecting the hub B, observing the conflicts during arrivals and departures. Observing trajectories over the already congested hub (risk of conflicts). Zooming in and out to observe locations of conflicts and the precise locations of trajectory lines
+00:01:33	PerceptionPoint	Implement	Inspecting hub A. Observing the risk of traffic crossing the already congested takeoff and landings. The operator can see that the conflicts during start and landing is within-service, but that trajectories could occur over it
+00:01:33	DecisionPoint	Effects	Effect goal, no conflicts between services over hubs
+00:01:33	DecisionPoint	Values	Deciding that the amount of trajectories observed in the simulation, that could cause conflicts, justifies an intervention
+00:01:33	DecisionPoint	Frames	Framing the situation as a between-conflict issue over the hubs
+00:01:36	DecisionPoint	Values	Deciding that this amount of conflicts justifies an intervention
+00:01:36	PerceptionPoint	Implement	Inspecting the conflicts between crossing trajectories between the hubs and their services
+00:01:47	ActionPoint	Generic	Adding service-specific geofences for both hubs, allowing only the own service traffic inside
+00:01:56	ActionPoint	Implement	Adjusting the geofences to the specific places
+00:02:08	PerceptionPoint	Implement	Checking the effect of the geofences by fast-time simulation. Zooming out to see the whole, zooming in to see details
+00:02:13	DecisionPoint	Effects	Effect goal, no conflicts between services in the space between the hubs
+00:02:14	DecisionPoint	Values	Again, judging whether amount of conflicts justifies an intervention vs. the effect goal
+00:02:14	DecisionPoint	Frames	Framing the situation as a between-service conflict, recalling that they use the same default altitudes going in and out
+00:02:15	PerceptionPoint	Implement	Observing continued problems with conflicts between services
+00:02:22	PerceptionPoint	Implement	Closer inspection of the conflicting trajectories
+00:02:40	ActionPoint	Implement	Adjusting the flight levels of service A
+00:02:54	PerceptionPoint	Implement	Observing the immediate effects, solved conflict
+00:03:06	PerceptionPoint	Implement	Testing with fast-forward, checking that no new conflicts emerge
+00:03:23	DecisionPoint	Frames	Deciding the services are de-conflicted

In step *iii*, we divided the Score into episodes of diagnosis (to understand the need for control interventions) and control activities to achieve effect goals by setting different constraints (using airspace structure elements, such as layers and geofences).

In step *iv*, the joints placed in step *ii* were scrutinized and adjusted with respect to the LACC levels. To decide on the degree of automation, for each control activity, we must also ask if it could and should be automated.

### Evaluation and Validity Procedures

Two validity procedures were employed in this work: Investigator triangulation and respondent validation. Investigator triangulation was used for problem situations that were cooperatively framed by two of the authors of this paper. The design iterations were created by one of the authors, and they were complemented by claims analysis and further ideation by the others. Respondent validation was used as scenarios were addressed with developers and with respondents in a claims analysis workshop (see procedure above).

### Research Ethics

No sensitive personal data (race, ethnicity, political views, religious beliefs, union memberships, health, sex life, or sexual orientation) and no personal data about criminal offences were recorded in the workshops. No physical procedures and no methods that aimed to affect the participants physically or psychologically were used, and no research was done on biological material from people. This means that no ethics approval was required from the Swedish Ethical Review Authority. Also, the researchers had no connections to commercial interests in the area, information about the study was given to the participants in advance of the workshops, and all participants filled out consent forms before the workshops started. Pseudonyms are used for all participants in transcribed material to ensure a certain degree of anonymity.

## Results

The results are described following the scenario-based design process. First the envisioned problem themes that were identified in Workshop 1 and the stakeholders and basic design assumptions are described. The key scenario for future operations is then presented. This is followed by the claims made by air traffic controllers in Workshop 2, and finally there is an analysis of temporal aspects of the cognitive work performed in the joint human–AI system.

### Envisioned Problem Themes

In total, 19 potential problem situations were identified in Workshop 1, and they were categorized into four themes: Regular traffic situations (e.g., arriving and departing aircraft, drone taxis and delivery drones), disturbances (e.g., noise pollution, weather, solar flares, and rouge drones), scheduled events (e.g., a football game at the stadium), and unscheduled events (e.g., fire and police action). A recurring theme in the workshop was a reference to “The Computer in Brussels”, which is a metonymy referring to a centralized, automated processes that they, as air traffic controllers, do not have control over. Instead, they just follow what that centralized traffic management system tells them, thereby realizing an example of human autonomy relinquished to automation.

### Stakeholders and Basic Design Assumptions

The UTM operators were the primary stakeholders for the UTM system design. Secondary stakeholders who depend on their work include priority stakeholders (e.g., police and emergency healthcare), drone service operators that fly the drones, companies that use the drone services, regulating authorities, and the local government. The citizens are tertiary stakeholders impacted downstream by the activities of all the primary and secondary stakeholders. All these stakeholders need to be addressed to create feasible, desirable, viable, and sustainable UAS-based services. Workshops with secondary and tertiary stakeholders of the UTM system are reported elsewhere (Lundberg et al., 2018a; Lundberg et al., 2018c; Halvorsen, 2018).

The stakeholders form a value network and bring different resources to the services provided and they strive for different values. The system under design aims to facilitate in the integration of the resources that the stakeholders bring to the network (Overkamp et al., 2018). UTM operators strive to resolve traffic situations through plans that can be implemented without undesirable side-effects for other stakeholders; priority stakeholders to be able to cut in line or to get privileged access to an airspace; the companies that use drone services can be expected to care for their brand and thus want to provide the service to their customers as promised as efficiently as possible; the regulating authorities set the scope and regulations of operations, and the local governments decides the rules for traffic in their municipality.

A basic design assumption for our work is that positioning and steering (detect and avoid), to keep operations of single drones within decided limits, is managed by drone operators and technology providers. It is also assumed that free flight is desirable, within the limits of restrictions, and that a minimum of regulation also is desirable.

### Key Scenario for Operations

Table 2 presents the selected key scenario for operations. This scenario describes the envisaged work activities of Sam, a hypothetical air traffic controller, as she places traffic components in the airspace and makes modifications to them.

**TABLE 2:** Key scenario. See Figure 1 for a visualization elaborating on part 3.

Part	Scenario
1	The public transport service has had a few drone passenger transportations between the train station and the airport, and there has also been parcel deliveries to the airport. The delivery truck has made a delivery using 15 drones in haga, during which a no-fly zone for other drones was automatically set up. One drone had to wait outside that zone and landed at an automatically assigned landing spot to not run out of battery.
2	A few regular scheduled aircraft have landed and taken off at the airport for which the scheduled geofences went up and down as planned. Sam can hear on the radio that the ambulance helicopter is approaching the vrinnevi hospital, so she sets up a temporary geofence for the landing. The drone traffic is heavy, so she sets a clockwise direction at the bottom level of it, and a counterclockwise direction to the top level. This means that traffic should flow smoothly around the hospital during the ambulance helicopter landing and take-off.
3	Now, during the late afternoon and early evening, there is quite heavy traffic to and from the two large delivery hubs in the slottshagen area. People want their packages delivered now that they are home from work. Many drones from the two closely situated hubs have intersecting paths, causing a lot of detect-and-avoid occurrences, which results in an inefficient airspace. Sam positions a landing zone over each of the two delivery hubs. The landing zone implements a selective geofence for other drones than those that belong to the operator who owns the hub. There are still conflicts between the operators, but she solves that by assigning the two operators to different flight levels.
4	Tonight, there will be a football game at the stadium, so sam needs to remember set up no-fly zone for that too. She could assign the TV company’s drones to the bottom layer and assign all other drones to the top layer. Instead, she decides to place a selective geofence over the football stadium where only the drones from the TV company can fly.

Besides from this key scenario, there were also other scenarios developed but not included in this article. They described how Sam could use global settings for the entire airspace, and work with a tube grid concept, routing the drones along pre-defined road network. There were also scenarios describing the work of a super user who built re-usable traffic components for the airspace and tried out new ideas for how to structure the airspace.

**FIGURE 2:**
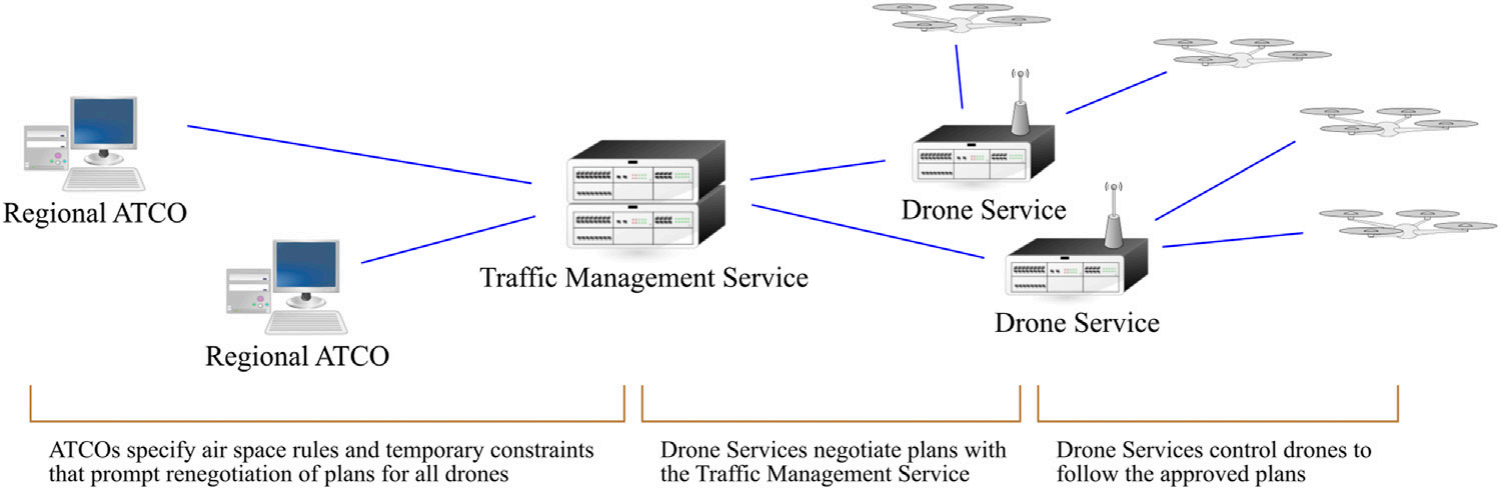
A generic UTM architecture. ATCO is short for air traffic controller.

The key scenario focuses on the left part of the generic system architecture depicted in Figure 2. It is a simplification of the more detailed architectures for drone operations that are currently (as of 2021) being developed. For instance, in Europe there is the U-space concept of operations. The scenario concerns what in U-space is referred to as drone automation level U4, which still is treated as exploratory (Sesar, 2019). U4 corresponds to the US UTM TCL4 (NASA, 2018), which is the most fully developed stage of UTM operations in cities. Figure 2 is comparable to the U-space system breakdown described by SESAR JU (2019, p. 83).

For the purposes in this paper, we can describe the architecture in Figure 2 in terms of LACC. Level 6 framing includes pre-conceptions of for instance the need to manage the airspace centrally, or that some stakeholders such as local government must be able to set airspace goals for their city, and that drone traffic must be kept within limits. Such frames apply to the entire UTM system but are embodied in the *Traffic Management Service*. Level 5 effects are what stakeholders aim to achieve and goals set in local regulations. They are embodied in *different parts of the system* depending on the stakeholder. On the one hand, the *Drone Services* embody the goals of the drone operators and negotiate these with the *Traffic Management Service* that embody national and international regulations. On the other hand, the local regulations and temporary constraints are embodied by the *Regional ATCO*. Level 4 values, such as risk levels, congestion levels, disturbance levels (e.g., noise) are set by the *Regional ATCO*. Some values may be open for negotiation while others are not. Level 3 generic plans, include structures of geofences, and intersection models for route networks. They are in our scenario set and monitored by the *Regional ATCO.* In contrast, the Level 2 implementation of them is done by the *Drone Services*. Level 2 implementation also includes the supporting systems of the *Traffic Management Service* such as certain communication, navigation, and surveillance systems, and specific ways of enforcing for example speed limits. Level 1 physical refers to the direct control and steering of the drones in relation to physical environment aspects such as ground and air hazards in specific areas, the physical location of all drones, their landing spots, and the physical aspects of geofenced no-fly zones. The *Regional ATCO Service* needs to be aware of processes at this level, but it is directly managed by the *Drone Services*.

The scenario design was followed by information and interaction design. That part of the process is not in focus for this article. However, the design questions used there focused on bridging level 3 and level 2 in LACC: what generic schemes (re-usable functions, plans, structures and patterns) should be included in the design, what should the operator do with them (level 2), and how should the user interface be presented for the UTM operator (level 1).

### Claims Made by Air Traffic Controllers

In Workshop 2, the key scenario (Table 2), was read to the participating air traffic controllers. This led to a “what if” discussion about the appropriateness of the different alternatives in relation to their needs. In the following, “level” refers to Level of Cognitive Control (LACC).

One issue that was discussed at some length was how to create a no-fly zone around the low flying approaching ambulance helicopter (Table 2, part 2), and what would happen if it landed not at the hospital but somewhere else (level 6 framing and level 5 implicit priority). Suggested design alternatives included moving geofences, stationary temporary geofences, and dedicated tubes (level 3). The choice would have to depend on other actors’ ability to comply (level 2). Another issue discussed was whether the human UTM operator would have time to react and act. If not, the conclusion was that it must be automated or delegated (level 2 operator). It was also discussed how priority is decided and by whom, and it was clear from the discussion that no model or principle for priority has been set (level 4). Finally, there was a discussion on how to measure airspace quality and safety (level 1–3) and what the KPIs are (level 4). Overall, the themes circled around what drones that are given *priority* and why; how *reliable* and safe the drones are including where they should crash upon malfunction; the *degree of automation* and the human operator’s role in the system; and finally, the balance between *centralized* and *distributed* planning and control.

The scenario in Table 2 also included the organizational principles for air traffic that both the operator and the system work with, i.e., temporary geofences (level 3) and landing zones that would work like geofences or dedicated vertical tubes (level 2). This frames (level 6) the work in terms of specific materials and processes that the human and automation both should work on. As we shall discuss, freedom of choice in working with different concepts is a key aspect of human autonomy. The participants discussed implications of different concepts for their ability to control traffic. It was appreciated that the geofences were dynamic and temporary rather than absolute and permanent, which meant that they could be planned for (level 3). On the negative side, it was noted that regular traffic changes and is never exactly on time. Thus, there is always need for re-planning (level 2 and 3). Having layers for different traffic directions was seen as easy to implement (level 2), and geofences could also be made more efficient by separating the traffic around them depending on direction. It was also noted in the claims analysis that it would be possible to separate traffic depending on the kind of traffic (passenger transports at higher layers and small drones at the lower layers) or operator (in the case of traffic from two delivery hubs that intersected). It was also noted that zip-merger (i.e., vehicles merge from multiple lanes to one lane by alternating turns) could reduce traffic jams and that it should be included in the general or global rules and settings for the airspace.

### Joint Human–Artificial Intelligence Control in Temporal Cognitive Work

Timing and time were important issues in Workshop 2, and we therefore made a more detailed analysis of temporal aspects, through an interactive simulation. We focused this analysis on the part 3 (Table 2) of the scenario. In this scenario the overarching frame is that control is centralized to what air traffic controllers in Workshop 1 referred to as “The Computer in Brussels”, that coordinates and plans the traffic.

**FIGURE 3:**
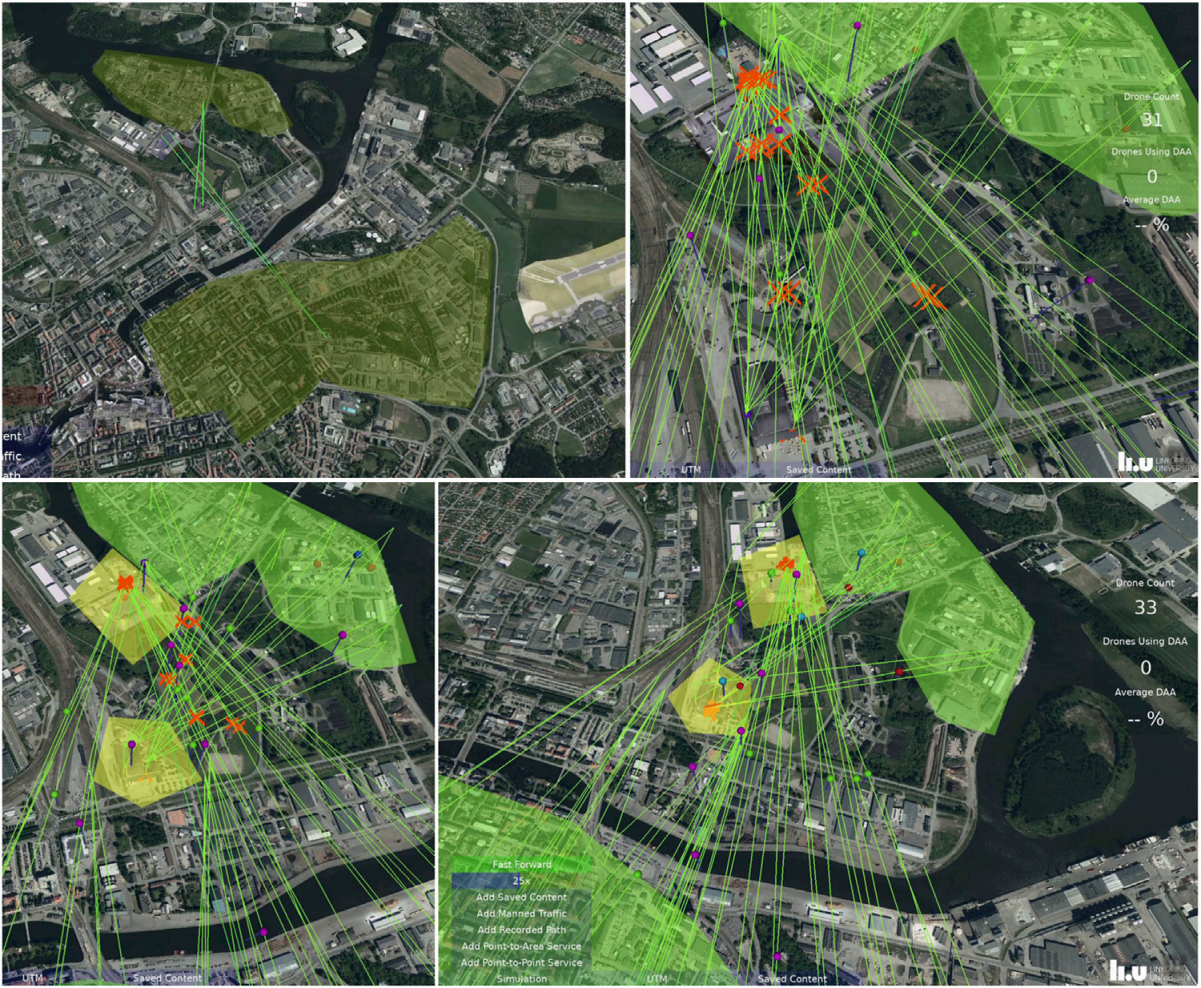
**(A)** (top left) service starts. Service areas in light green, closest path from a hub to its delivery area in green lines. **(B)** (top right) fast-forward shows conflicts. **(C)** (lower left) hub start/landing locations de-conflicted (the two lines that go across the geofence are the shortest-path lines, not traffic), **(D)** (lower right), crossing traffic in the airspace de-conflicted. Background generated from GSD-Ortofoto25 and GSD-Höjddata, grid 2+ **(C)** Lantmäteriet.

To allow continued analysis of this scenario with respect to temporality, it was implemented in our interactive drone traffic simulator, see Figure 3. In the following, we are concerned with this system as a tool for understanding, deciding, and acting on the traffic. The scenario starts with two services that are pre-approved from a ground risk perspective. As the scenario starts, their interaction with other services and other traffic is the main concern. We played this scenario (and variations of it) several times, resolving the issues that became visible. As this scenario plays out, the first temporal concern is when the analysis and de-confliction should occur. Should deconfliction occur when problems emerge in the traffic situation (as in the original workshop scenario) or before the service starts? The underlying problem with the original scenario is that the conflict situation between the two operators has already happened. Instead, it would be useful to use simulation as a planning tool before the drone operators are authorized to start. In the scenario analyzed using the JCF Score (Figure 1) the scenario is thus instead conceived as a planning task that is done after the two delivery services have been approved but before they have started flying. This could be near real-time. We will now show an analysis of how that work would take place. It contains the same activities as the original scenario presented above. The only modification is that the traffic has not yet started, and it will be simulated before final approval.

If we analyze the overarching temporal structure of this simulation, we find that the scenario must start with inspection of the service and airspace structure, then proceed with adding geofences around the delivery hubs to exclude crossing traffic, and finally deconfliction of the en-route segment by altitude. This reflects three frames (LACC Level 6, L6). We also find that the air traffic controller uses fast-forward to be able to inspect the fully developed traffic situation (e.g., at 01.06, yellow segment of the scenario in Figure 1 and Table 1). In this scenario, the simulation shows predictive modeling of conflicts (L2), which makes the conflicts visible before occurrence in the simulation, for the planned routes. The operator can thus base decisions both on the occurrence of crossing trajectories and predicted conflicts at those crossings. The operator moreover uses fast-forward to inspect locations and recurrence of conflicts over time. We can see that the operator mainly works on Level 3 and Level 2. The operator inspects generic plans for those two services, adding generic geofences for the two hubs (L3), and then adjusting them (L2) to the situation. The operator all the time works from particular and developing frames (L6). The effect goals (reduce conflicts, L5) are associated with these frames. The initial frame can be seen as associated with few/no conflicts as a core value at level 5, rather than as a decision in the episode. However, the operator judges (L4) the number of conflicts vs. the goals (L4, values) vs. the effect goal. This judgment leads to a decision on whether to proceed with the green and blue parts of the episodes (de-confliction). All in all, it took 3 min and 23 s to run this in the simulator and reach the point at which a specific plan and rules for the delivery services can be set.

## Discussion

This paper reports a case study of scenario-based design for human autonomy in interaction with artificially intelligent autonomous systems. We will in the discussion below relate the results more closely to the levels of abstraction in the Levels of Autonomy in Cognitive Control (LACC) model, and the Joint Control Framework (JCF) (Lundberg and Johansson, 2020).

### Human Autonomy on the Levels of Cognitive Control

Our analysis suggests that it is too simplistic to say that there either is human autonomy, that is, the freedom to reason without constraints from authority and preconceptions (Stensson and Jansson, 2014), or autonomous systems that impede on that human autonomy. Our analysis shows that restrictions and autonomy enablers in this case do exist in the human-autonomous system relations, but also emerge from the larger sociotechnical system and between humans. We will present our analysis supporting this based on the LACC, starting with autonomy in framing of situations (level 6).

Level 6 concerns the autonomy to frame situations. Systems we build can corner us and limit our ability to act on new frames and re-conceptualizations. That is, autonomous systems will limit what humans can do, but also what we can imagine doing. We must however also recognize that autonomous systems, and simulations in our case, also support our ability to act, as well as our ability to collectively imagine what to do. Automation is a double-edged sword; it always enables as well as constrains human autonomy. For example, in our second workshop one participant introduced a new frame of a low flying approaching ambulance helicopter (Table 2, part 2), that also might land, not at the helipad, but somewhere else. This created a provocation to the design principle of free flight in our initial concept. This principle and frame is reflected also at lower LACC levels. An alternative solution within the frame of that concept was to have a moving geofence or a stationary temporary geofence. However, if our simulation had been built on the principle of a grid of tubes, it would have been easier for the air traffic controller to suggest, and for the system designers to choose the alternative of a dedicated tube for the ambulance helicopter. The frame thus reflects on concepts of planning at level 3, implementation at level 2, and objects at level 1. If the Computer in Brussels is built around the frame of free-flight, and lacks the notion of tubes, this limit human autonomy in control–even in a case where the human suddenly sees the need for tubes through a re-framing of the situation. The autonomy in going from frame to control at lower levels is in this case as important as the autonomy in framing per se. The framing made at one time limits the autonomy of introducing new frames at another time. The frames and principles behind the implementation of The Computer in Brussels sets restrictions on both what we can do and what we might imagine doing. The processes we have noted in this case involves what Schön (1983) writes of as the narrowing down of choice through framing.

Level 5 human autonomy concerns who it is that has the mandate and the ability to set effect goals. An autonomous human decision for one actor might imply a restriction of autonomy for other actors. For the overall system to work and manage conflicting goals, some people will have to comply with the goals set by others. The goal of efficient deliveries for a drone operator may for example have to yield for the goal of reducing noise pollution set by the urban planners. Potentially, the Computer in Brussels could also introduce a new goal into the situation, impeding in the autonomy of the human actors to set their own goals. Thus, the human autonomy is also always relative to the mandate you have. In the case of deciding the goals, there will always be human actors as well as autonomous systems that do not have the autonomy to make the choice. Conflicting goals may require trade-offs (level 4), but the more overarching issue at level 5 is the autonomy to decide what effect goals to pursue. Further, with mandate comes also accountability, and the responsibility to set goals that others can follow. That then becomes an issue of implementation, and the ability to consider what can be achieved at lower levels when setting goals. An example of this from our case is when the ambulance helicopter (Table 2, part 2) makes its approach and drones need to move out of the way. First, regardless of whether the human or the system attempts to move the drones, there is a limit to how quickly they can respond. Further, if the movement is to be managed by the Computer in Brussels, but it does not have the procedures in place (or the means to plan) to realize the goal, then this places a limit on the autonomy of the human. If the movement is delegated to the autonomous system, it may also affect human autonomy in making some drones exempt from the movement.

Level 4 human autonomy is about the tradeoffs between conflicting goals and who it is that makes the tradeoffs between competing key performance indicators (KPIs). For example, a design issue in our case is if it is the air traffic controller who decides the tradeoff between noise level and efficient deliveries, if it is the drone operator, or if it is The Computer in Brussels. On a more general level, this refers to how priority is decided and by whom. A related issue is if there are methods to measure those KPIs. It will become a limiting factor for autonomy of both humans and AI if there are no such methods available, or if measures or approximations are not done in the same way by everyone involved.

Level 3 human autonomy addresses the ability to plan and change already made plans. Here the human ability to catch up and re-plan in the face of a changing situation will be a limiting factor, especially for control centralized to The Computer in Brussels of not only ten, but hundreds of drones. For example, it was perceived as positive in the claims analysis that geofences could be planned to go up and down, instead of having an absolute and permanent traffic situation. However, in the case of the quickly approaching ambulance helicopter, will there be time to change already made plans? In what situations are there room for human autonomy in the planning activities at level 3? The already-made building blocks (e.g., a temporary geofence) are pre-constructed plans that the air traffic controller can use to re-plan, which leads us to the next level.

Level 2 human autonomy involves situational adjustments and how the autonomous systems are built. In our scenario (Table 2), the air traffic controller placed and made variations of the pre-defined traffic management solutions (in the form of building blocks into the airspace) to make such situational adjustments. This requires that the automation is open to human intervention, that is, that it presents an interface to see and manipulate at level 2. There is also a question of if the human or AI agents piloting the drones can comply with sudden changes to plans. This is a limiting factor for the autonomy of the air traffic controllers to manage the airspace as they see fit. This was seen as an issue for drone operators in our claims analysis, but it was also an issue for regular air traffic controllers since the plans made for regular aircraft also are subject to sudden changes.

Level 1 human autonomy concerns the ability to perceive the status of an object and affect it. This is at the level of tracking and steering actions. It involves driving each drone manually as every person sees fit, and the autonomy to follow or violate regulations and constraints set up by an air traffic controller. This also relates to the controls offered from the drones to human pilots, and the levels of skills and abilities needed for the pilot to manage those controls.

This analysis of autonomy at the different levels of control leads to a design decision made already in the core concept. It builds on the premise that there will be too many movements in the air at once for the air traffic controllers to be able to go in and manually control them object by object. We thus opted for not giving the air traffic controllers autonomy on Level 1. This gives them instead the autonomy to work on primarily level 2 and 3. They do not have full autonomy on the levels above (4–6) since they are governed by rules and regulations. For a person concerned with effect goals and making decisions between them in the scope of action provided by the system, autonomy at levels 4–5 may be the most important. A person in this role would plan the traffic and set the rules for the airspace. Having the autonomy on a higher level of control can restrict autonomy at lower levels since framing, regulation, and planning restricts what can be done in operations. However, autonomy at a lower level can also prevent autonomy on higher levels: if the air traffic controller would have the autonomy to steer every movement drone by drone, there would not be any time or cognitive capacity to think ahead, plan or re-frame the understanding of the situation. Working on different control levels also requires different skills, practices, and habitual ways of thinking and acting. For a human who nevertheless have the skill and wants to enjoy more direct control and immersion in the on-going process, roles might shift so that the automation becomes the overseer of human action. Consequently, autonomy is dynamic and subject to change across control levels. If the human only has autonomy on level 1 (i.e., steering), then the human is largely controlled by the automation. In contrast, if the human is guided by an automation working at level 4, this could provide feedback on the qualities (KPIs) of the human actions, while still enjoying a great deal of freedom on how activities are organized, implemented, and acted on. Further, the human could shift between high- and low-level control. However, due to time limits, the human operator may not in all cases be able to work at all levels at the same time, and not with all the low-level control activities that are needed to steer a whole system. Finally, human autonomy at level 6 can make the use of autonomous systems problematic since a dramatic change in framing can require equally dramatic changes in the required abilities of the autonomous system. Thus, the autonomy to work with effect goals (at level 5) may come with a loss of autonomy at lower levels.

### Temporal Aspects of Autonomy

If we turn from the levels of LACC to the temporal dimension of the scenario (Table 2, part 2) in the JCF Score illustrated in Figure 1 and Table 1 we can observe a pattern of action and reflection that is central to our discussion on human autonomy. The pattern is one of high-level valuation and assessment, followed by action on lower levels (generic, implementation), and then inspecting the situation primarily on the implementation level, before finally zooming out and reflecting at a higher level again (levels 4–6). The scenario starts with a core value (an efficient and effective airspace) that the human operator brings to the situation. With that as the opening frame, initial actions or moves are made. The operator then observes simulated effects of the moves through the backtalk (Schön, 1992) of the simulation. The operator then zooms out, questioning and filling in the initial frame with details, then performing new actions. This pattern repeats itself four times in the episode. The process has no clear-cut start or ending, but we will discuss each of the parts of this pattern.

In the reflection part of this pattern, backtalk from the situation provided by the simulation (what can be seen, i.e.,) is central. There is on the one hand the dynamic aspects (routes, drones, conflicts) and on the other hand the more static map information. The simulation in this case helps with understanding of the particular, at control levels 1–3. It shows, however, no information on higher control levels (4–6). The system gives a strong signifier (Norman, 2008), to address the red crosses (i.e., the conflicts) by restricting the traffic. Though, the means for that are not directly perceivable. Resolution of conflicts in this scenario takes place through what Winograd and Flores (1986) and Schön (1992) would describe as a conversation with the simulation.

The perception (backtalk) part of the pattern is linked to the framing of situations (Schön, 1992). The Score shows that the controller initially brings with them a core value, which is organizational in nature. This core value restricts human autonomy as well, by limiting what is perceived as appropriate actions. The simulation also restricts human autonomy, through the same frame, by the focus on conflicts and routes. Regarding the underlying map of the simulation (the aerial image), it does not show much useful information for the framing in this scenario. However, changing the map would affect human autonomy, limiting or facilitating the human ability to take other frames. Within the frame, simplifying the map would probably even be beneficial to make it clearer and more efficient. Adding data layers on, for example, crowds or noise, would affect the human ability to take other frames (i.e., considering safety on the ground instead of conflicts in the air). Accordingly, making a more efficient system within one frame can limit the human autonomy to make new frames or question the current frame.

Turning to action, the AI has a significant impact on human autonomy. It gives leverage points for the operator to make higher-level control decisions (e.g., setting goals, metrics, and generic schemes), and make decisions that are aimed at groups of drones (e.g., an entire service). The human autonomy is limited to the means provided by the AI, for example, adding geofences to re-direct traffic flows or changing flight levels or speeds, but not addressing individual drones. Further, when the services have been planned using the simulation, the authorized plans has to be followed, at which the autonomy of the service providers is restricted.

The temporal aspect of autonomy relates to a fundamental design question: when should the human operator act? It can be during planning, before a drone service has started, or later when the drone service is in operation. If the design is to act as late as possible, then this would also restrict human autonomy, since there is less leeway for actions to be taken during operations. There would also be less time for framing and re-framing the situation, restricting the autonomy even further. An autonomous AI system that takes care of operations can therefore increase human autonomy by making it possible to plan earlier in advance and hence give increased freedom to reason without constraints from authority and preconceptions.

Further, to decide on the degree of automation, for each control activity, we must also ask the question–couldn’t the computer in Brussels do this as well? It can be tempting to remain in one framing (e.g., that the situation is a conflict situation) and try to resolve that without questioning whether it is the “right” framing. Then it might be inviting to also optimize for that, and perhaps push the human out of the loop more (the computer doing more of what the human now does at Levels 3–6). This optimization can be done during system design, or incrementally, by for example adding heuristics or machine learning techniques. In an extreme case, the human is pushed out of the control loop completely. For the human then to re-gain some autonomy, the AI decision-making process must be opened up by some means of transparency, that the human can see. It must also be opened up through new leverage points, for the human to affect it. Thus, the direct control in the scenario might play out much in the same way, but with inspection and reflection shifting to the automation. For the human however, it would change completely, with other points of inspecting and affecting the AI.

To conclude this discussion on temporal aspects of autonomy, we argue that decisions made at one point in time on one level will limit the autonomy of not only yourself but also others at a later stage on another level, which, again in Schön’s (1983) terminology, implies that any such framing narrows down subsequent human autonomy. On a large timescale, this includes decisions made already during the framing and design of the system that have an impact for the autonomy of different actors when the system is put to use. On an intermediate timescale, this has to do with the regulations, goals, and KPIs that are set in regulations, policies, and guidelines. Finally, on a small timescale, it relates to how an UTM operator limits and facilitates the autonomy of drone operators and pilots in the airspace, and how they limit and facilitate the autonomy for each other.

### Limitations

The fidelity of the simulation is on a medium level. The drones traffic behaves realistically but with some exceptions, and the scenarios are at a low fidelity level. This means that the representational validity can be said to be at medium level (Feinstein and Cannon, 2002). The responses from air traffic controllers in workshops indicated that the scenarios and events in the simulation were perceived as realistic, which implies that there is internal validity, which in turn makes application validity attainable (Feinstein and Cannon, 2002). The results have, however, not been field tested and we do not present a verified solution on how to design a UTM system. Scenarios were tested in a workshop and in a simulation, but we do not yet know how well our results transfer from simulation to practice, and the external validity remains therefore to be verified. Also, this paper does not present all scenarios and design solutions developed, due to space restrictions, and the included scenarios do not cover every conceivable aspect of a future context of use. They are instead used as material to theorize the concept of human autonomy in interaction with autonomous systems. To this end, we have used the joint control framework and levels of autonomy in cognitive control model as vehicles for abstraction from the case to a theoretical level, which then can be used in new cases.

### Future Research

Case studies are difficult to replicate in the sense used in for example experimental research. A similar case can however be selected for a subsequent study to see if it yields similar results regarding human autonomy. Such case studies could use a similar design-modelling-simulation-analysis approach, with a comparable descriptive framework, and focus on networks of actors (human and artificial) and their power relations during episodes of control. Another direction for future research could be to explore operator interfaces that automatically highlight autonomy relations to make the operator aware of who has what autonomy.

Other theoretical frameworks would naturally focus on other aspects of designing for human autonomy (e.g., abilities, permissions, accountability), and the impact of those aspects is a topic for future research. The choice of theoretical framework is hence a meta-framing of the design effort and it sets what Hult et al. (2006) call a *design perspective*. Changing perspective can drive divergence in a design process, as well as provide multiple perspectives on what is valuable while also highlighting aspects such as power relations between AI and human actors or bias (Arvola and Holmlid, 2015; 2016).

Another area of future research is methodological in nature. The design and analysis work in this paper builds on JCF and the HMT-T Score notation, and it makes a limited methodological contribution by providing an example of how JCF can be applied in systems design. No guide to the design of joint human-AI work is however provided here. That is instead a topic for another paper.

## Conclusion

This paper has contributed with a description of how to design and reflect on human autonomy in interaction with artificially intelligent and autonomous systems, by means of scenario-based design structured by the abstraction hierarchy in the Levels of Autonomy in Cognitive Control Model and the temporal aspects of the Joint Control Framework.

These theoretical frameworks have facilitated the discussion of human autonomy in the UTM system design. It has helped the research and design team to see what we have covered and how it connects different system elements. The simulation is the mechanism that drives the design work and makes it concrete, and therefore available for reflection.

A key issue is how we define human autonomy. The UTM controllers are still autonomous persons if they get to set the goals, regulations, and plans on a higher level, even if they are not allowed to make situational adjustments or steer. If they instead had the autonomy to steer and make situational adjustments, they would not have time or cognitive capacity to plan ahead and re-frame their understanding. Hence, if the UTM system is automated so that controllers can work effectively on the higher abstraction levels, like planning and regulating instead of steering, then we can argue that we have increased human autonomy (i.e., the freedom to reason without constraints from authority and preconceptions). This is a design and engineering trade-off between technological opportunities and what system designers, policy makers, and users find valuable and meaningful. The freedom to choose for yourself what levels of cognitive control that you would want to be autonomous on, will however always put limitations on the freedom to choose for others. The design of autonomous systems is thus inherently a question of power.

## Data Availability

The datasets presented in this article are not readily available because of anonymity of the participants. Requests to access the datasets should be directed to registrator@liu.se.
